# Human mesenchymal stem cells derived exosomes improve ovarian function in chemotherapy-induced premature ovarian insufficiency mice by inhibiting ferroptosis through Nrf2/GPX4 pathway

**DOI:** 10.1186/s13048-024-01403-6

**Published:** 2024-04-15

**Authors:** Yuan Zhou, Jinfa Huang, Lingling Zeng, Qian Yang, Fangjuan Bai, Qiqing Mai, Kaixian Deng

**Affiliations:** Department of Gynecology, Shunde Hospital, Southern Medical University, Foshan, Guangdong 528308 China

**Keywords:** Chemotherapy, Ovarian dysfunction, Exosomes derived from stem cells, Ferroptosis, Nrf2/GPX4 pathway

## Abstract

**Background:**

Chemotherapy exposure has become a main cause of premature ovarian insufficiency (POI). This study aimed to evaluate the role and molecular mechanism of human umbilical cord mesenchymal stem cell-derived exosomes (hUMSC-Exos) in ovarian function protection after chemotherapy.

**Methods:**

hUMSC-Exos were applied to cyclophosphamide-induced premature ovarian insufficiency mice and human ovarian granulosa tumor cells (KGN) to determine their effects on follicular development and granulosa cell apoptosis. Evaluation was done for iron ion and reactive oxygen species (ROS) production, lipid peroxidation levels, and changes in iron death-related molecules (nuclear factor (erythroid-derived 2)-like 2 (Nrf2), Glutathione Peroxidase enzyme 4 (GPX4), and Solute carrier family 7 member 11 cystine glutamate transporter (SLC7A11; xCT)). Furthermore, rescue experiments using an Nrf2 inhibitor were performed to assess the therapeutic effects of hUMSC-Exos on granulosa cells.

**Results:**

hUMSC-Exos promoted ovarian hormone levels and primary follicle development in POI mice and reduced granulosa cell apoptosis. After hUMSC-Exos treatment, the ROS production, free iron ions and lipid peroxidation levels of granulosa cells decreased, and the iron death marker proteins Nrf2, xCT and GPX4 also decreased. Furthermore, the Nrf2 inhibitor ML385 significantly attenuated the effects of hUMSC-Exos on granulosa cells.

**Conclusion:**

hUMSC-Exos inhibit ferroptosis and protect against CTX-induced ovarian damage and granulosa cell apoptosis through the Nrf2/GPX4 signaling pathway, revealing a novel mechanism of hUMSC-Exos in POI therapy.

## Background

Cyclophosphamide (CTX) is widely used in clinical treatment of tumors, inhibition of graft rejection after organ transplantation and immunosuppression of autoimmune diseases. However, a large number of women of childbearing age have a serious side effect after using CTX, that is, premature ovarian insufficiency [1]. Researches showed that up to 40% of women experience POI after chemotherapy, with a risk of lifelong infertility [2]. This has a profound impact on their quality of life and mental well-being [3]. Therefore, exploring new strategies to protect against CTX-induced POI is of utmost importance.

Exosomes are small vesicles with a diameter ranging from 30 to 150 nm, capable of carrying diverse bioactive molecules such as proteins, nucleic acids, and lipids. Emerging evidence indicates that hUCMSC-Exos participated in such physiological processes as cell proliferation, differentiation, migration, and apoptosis [4, 5]. Therefore, exosomes are considered as a promising therapeutic tool. One study showed that hUCMSC-Exos improved ovarian reserve and hormone levels in POI mice treated with CTX [6]. In addition, a preclinical study showed that exosomes derived from hUCMSCs enhanced the proliferation of granulosa cells and ovarian cells after CTX treatment, which may be related to a decrease in the accumulation of reactive oxygen species [7]. Consequently, this intervention partially restored the ovarian phenotype and function.

Iron plays an important role in cellular metabolism and survival, but excessive iron can induce oxidative stress, protein oxidation, and DNA damage, leading to ferroptosis and even cell death [8–10]. Recent studies have highlighted the significant role of ferroptosis in the treatment of chemotherapy-induced ovarian dysfunction [11, 12]. Chemotherapy drugs induce iron accumulation in ovarian tissues, leading to increased oxidative stress and damage to ovarian function [13]. Furthermore, excess iron can induce apoptosis in ovarian cells, affecting normal ovarian function [14]. Researches have shown that reducing iron accumulation, such as using iron chelators or iron channel inhibitors, can alleviate oxidative damage in ovarian tissues and protect ovarian function [11]. Additionally, interventions targeting iron metabolism pathways, iron absorption, storage, and metabolism, have been proved to mitigate the adverse impact of chemotherapy on ovarian function [15]. Whether hUCMSC-Exos can protect ovarian function by inhibiting ferroptosis remains to be explored.

In summary, this study aims to investigate the role and mechanism of hUCMSC-Exos in treating chemotherapy-induced ovarian dysfunction, specifically focusing on their potential to protect ovarian function by inhibiting ferroptosis.

## Materials and methods

### Culture and identification of hUC-MSCs

hUC-MSCs were obtained from Guangzhou DuDe Biotechnology Co., Ltd. They were cultured in α-MEM medium (Gibco, USA) supplemented with 10% fetal bovine serum from South America (Gibco, USA), 1% penicillin/streptomycin (Gibco, USA), and 0.05% bFGF. The fourth passage hUC-MSCs were selected and identified by flow cytometry. Cell surface markers including CD34 (Elabscience), CD45 (Elabscience), CD73 (Elabscience), CD90 (Biolegend), and CD105 (Endoglin) were used for MSC characterization.

### Identification of hUC-MSCs by adipogenic differentiation

The 4th-generation hUC-MSCs with a cell density of 80-90% were seeded into a six-well plate and cultured at 37℃, 5%CO2 and saturated humidity. When the cells reach 100% confluence, add adipogenesis induction medium A; replace it with adipogenesis induction medium B after 3 days of culture, and replace it with A after 24 h of culture. Repeat 4 more cycles. Add solution B and culture for 7 days, changing the medium every 3 days. Control cells grew normally. After 23 days of induction, cells were stained with Oil Red O. The cells were washed with PBS, observed and photographed under an inverted microscope.

### Identification of hUC-MSCs by osteogenic differentiation

Pluripotency assay was used to assess the pluripotent ability of MSCs to generate osteoblasts using commercially available differentiation media (StemPro Differentiation Kit, Thermo Fisher Scientific). To this end, different groups of mesenchymal stem cells were cultured in 6-well slides and evaluated histologically. Briefly, cells were cultured in osteogenic differentiation medium for osteogenic differentiation. Change differentiation medium twice weekly. After 21 days, differentiation evaluation and calcium deposition quantification was assessed by alizarin red staining.

### Isolation and identification of hUMSC-Exos

The 5th passage of hUC-MSCs, grown to 80–90% confluency, were cultured in α-MEM medium supplemented with 1% exosome-depleted serum and 0.05% bFGF for 48 h. The cell culture supernatant was collected and filtered through a 0.22 μm filter to remove cells, apoptotic bodies, and cellular debris. Sequential centrifugation at 300 g, 2000 g for 10 min, and 10,000 g for 30 min at 4 °C was performed to further eliminate contaminants. Subsequently, ultracentrifugation at 110,000 g for 70 min was carried out to isolate the exosomes. Finally, PBS was used for resuspending the exosomes. Nanoparticle tracking analysis was employed to determine the size of the exosomes, while transmission electron microscopy was utilized for exosome identification.

### Establishment of POI mouse model and treatment with hUMSC-Exos

6-week-old C57BL/6 female mice were purchased from Guangdong Yaoke Biotechnology Co., Ltd. (Guangdong, China). The mice were maintained at a temperature of 22 ± 1 °C, a relative humidity of 50 ± 1%, and a 12/12-hour light/dark cycle. Sterilized food and water were provided ad libitum. All animal experiments were conducted in accordance with the guidelines and regulations of Shunde Hospital, Southern Medical University, and the AAALAC and IACUC guidelines.

The mice were divided into three groups: control group, POI group (*n* = 10), and POI + Exos group (*n* = 10). To establish the POI model, mice received intraperitoneal injections of CTX (50 mg/kg) daily for 14 consecutive days, while the control group received an equivalent volume of PBS. In the POI + Exos group, mice were transplanted with 30 µl of hUMSC-exos (0.015 µg/µl) into each ovary one week after the POI model was established. Meanwhile, mice in the POI group were injected with an equivalent volume of PBS. At the end of the 2nd and 4th week after treatment, five mice from each group were euthanized under anesthesia, and serum and ovarian tissues were collected.

### Hematoxylin and eosin staining

The staining procedure for hematoxylin and eosin (H&E) was performed following the established protocol described in previous publications [16]. Briefly, ovarian tissues fixed with 4% paraformaldehyde (Servicebio, China) were dehydrated, embedded, and sectioned (The maximum transverse section of the ovary wasserially sliced into 4 μm sections). The sections were stained with hematoxylin and eosin. The morphology of each ovarian section was observed and photographed using an inverted microscope (Leica DMI1, Germany). The number of follicles at different stages was counted.

### Serum hormone levels determination by ELISA

Serum for hormone measurements was obtained by centrifuging mouse blood at 5000 rpm for 10 min. The levels of anti-Müllerian hormone (AMH), estradiol (E2), and follicle-stimulating hormone (FSH) in serum were determined using ELISA kits (Lengton, China). The optical density (OD) values were read at 450 nm using an ELISA reader.

### Culture and treatment of human granular tumor cells

KGN cells (Procell CL-0603) were provided by Wuhan Procell Life Science & Technology Co., Ltd. and cultured in DMEM/F12 medium (Gibco, USA) supplemented with 10% fetal bovine serum (Gibco, USA) and 1% penicillin/streptomycin (Gibco, USA). The cells were divided into four groups: the control group (Nc), the POI group (CTX, 1 mg/mL, POI), the hUMSC-derived exosomes (0.015 µg/µl) treatment group (POI + Exos), and the hUMSC-derived exosomes + NRF2 inhibitor treatment group (ML385, 1 µmol/L, MCE) (POI + Exos + ML385).

### EdU staining

Cell proliferation was assessed by EdU staining after both 24 and 48 h of culture in each group. Cells were treated with EdU working solution for 4 h, fixed with 4% paraformaldehyde, and then stained with EdU mixture and DAPI. Cell apoptosis was evaluated using Hoechst staining. After 24 and 48 h of cell culture, cells were incubated with Hoechst/PI staining working solution in the dark for 20 min. Finally, assessment was performed using a fluorescence microscope (Leica DMI8, Germany).

### Reactive oxygen species detection

The levels of reactive oxygen species in cells were measured using a ROS detection kit (Beyotime, Shanghai, China) following the manufacturer’s instructions. The probe for intracellular ROS (DCFH-DA) was diluted to a concentration of 10 µmol/L in serum-free medium. KGN cells were then incubated with the diluted DCFH-DA at 37 °C for 30 min. Fluorescence was detected using an inverted fluorescence microscope.

### Ferrous ion detection

A fluorescent probe for ferrous ions (FeRhoNox-1) detection kit (MKBio, China) was used to measure Fe2 + levels in cells. FeRhoNox-1 was diluted to a concentration of 5 µM in PBS (prepared fresh) and incubated with KGN cells at 37 °C for 60 min. Fluorescence was detected using an inverted fluorescence microscope.

### Cellular lipid peroxidation detection

A lipid peroxidation sensor (BODIPYTM 581/591 C11) (Shanghai BioHub Biotechnology Co., Ltd.) was employed to indicate intracellular lipid peroxidation and antioxidant status. Treated cells were stained with 5 µM BODIPYTM 581/591 C11 at 37 °C for 30 min. Fluorescence images of cells were captured using a fluorescence microscope.

### Western blotting

Western blotting was performed using the Multistrip Western blotting protocol as previously described [16]. The following antibodies were utilized: The exosomes were identified by CD63 (1:1000; Abcam), CD9 (1:1000; Abcam), TSG101 (1:1000; Abcam). The expression of Nrf2/GPX4 pathway was detected by Nrf2 (1:1000;Cell Signaling Technology), xCT (1:1000; Abcam), GPX4 (1:1000; Abcam), and Alpha Tubulin (1:200; Proteintech). After incubation with secondary antibodies at room temperature for 30 min and subsequent washes, the bands were visualized using enhanced chemiluminescence (Abcam, USA).

### Statistical analysis

All quantitative data are presented as mean ± standard deviation (*n* ≥ 3). Statistical analysis was conducted using GraphPad Prism 9.0 software, and significant differences were evaluated using one-way analysis of variance (ANOVA) and t-tests; *p* < 0.05 was considered statistically significant.

## Results

### Characterization of hUC-MSCs and hUMSC-exos

**Fig. 1:**
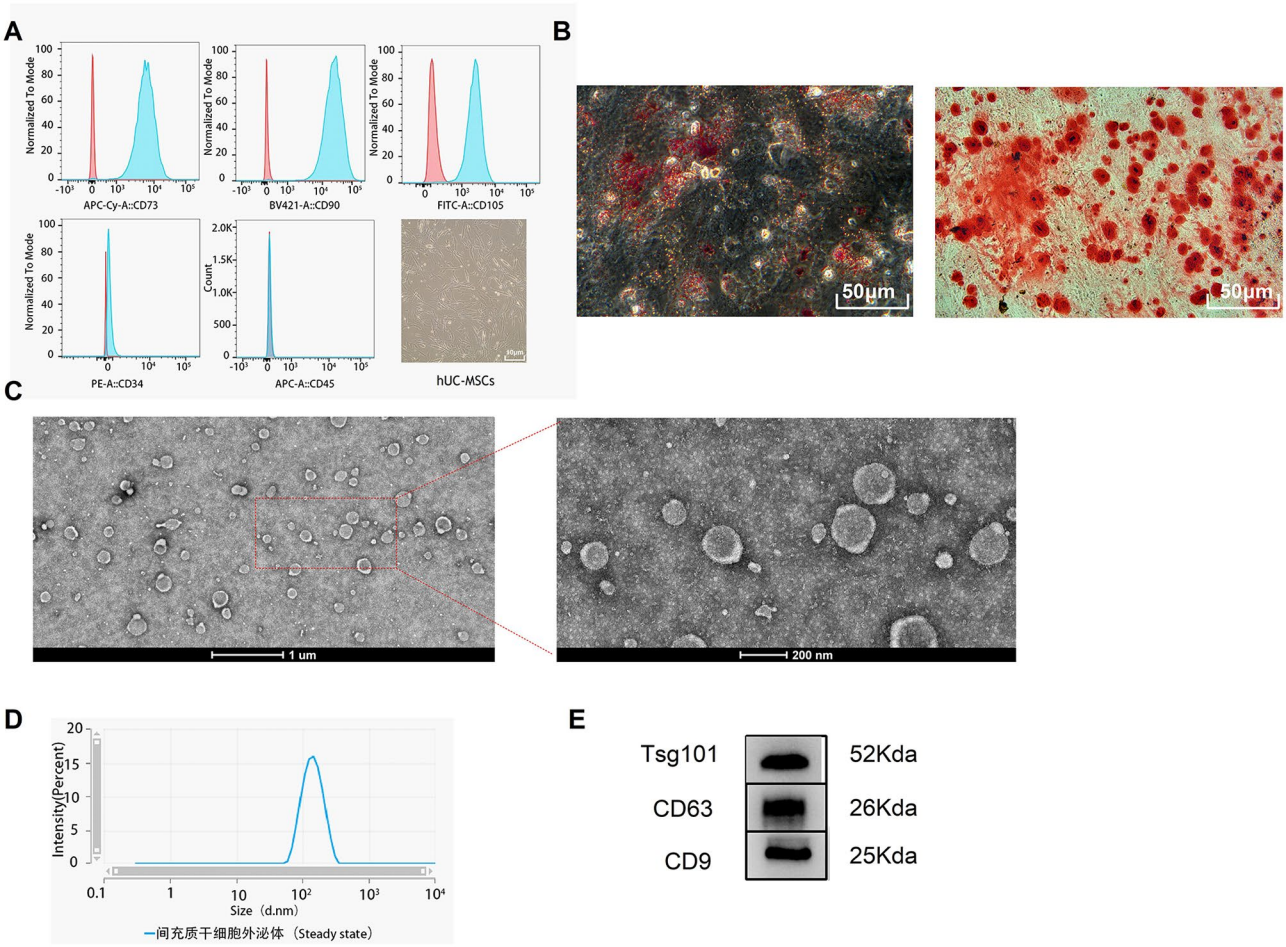
Identification of hUC-MSCs and hUMSC-exos. **A** Flow cytometry analysis of surface markers’ expression on hUC-MSCs and the morphological characterisation of hUC-MSCs under an optical microscope. **B** Osteogenic and adipogenic differentiation of hUC-MSCs. **C** Representative image of hUMSC-exos under Transmission Electron Microscopy (TEM). **D** Characterisation of hUMSC-exos via Nanoparticle Tracking Analysis (NTA). **E** Western blot identification of exosomal markers CD63, CD9, and TSG101

The human umbilical cord mesenchymal stem cells (hUC-MSCs) display spindle-shaped, fibroblast-like morphology. The MSC biomarkers (CD73/90/105) were positively expressed, whereas the haematopoietic and endothelial markers (CD34/45) were negatively expressed (Fig. 1A). Oil Red O and Alizarin Red staining demonstrated the ability of hUC-MSCs to differentiate into adipocytes and osteoblasts, respectively (Fig. 1B). Under transmission electron microscopy (TEM), exosomes exhibited the characteristic bilayer vesicle structure (Fig. 1C). The exosomes isolated from the serum-free culture supernatant of hUMSC-exos were characterised using TEM and Western blotting. Nanoparticle Tracking Analysis (NTA) revealed that hUMSC-exos appeared spherical with a concentration of 1.1 × 10^10 particles/ml and a peak diameter of 128 nm (Fig. 1D). Bicinchoninic Acid (BCA) protein assay indicated a protein concentration of 2.71 mg/ml in hUMSC-exos. Western blotting confirmed the positive expression of CD63, CD9, and TSG101 in hUMSC-exos (Fig. 1E).

### hUMSC-exos promote the recovery of ovarian function in mice

#### HUMSC-exos restored the ovarian function of POI mice

**Fig. 2:**
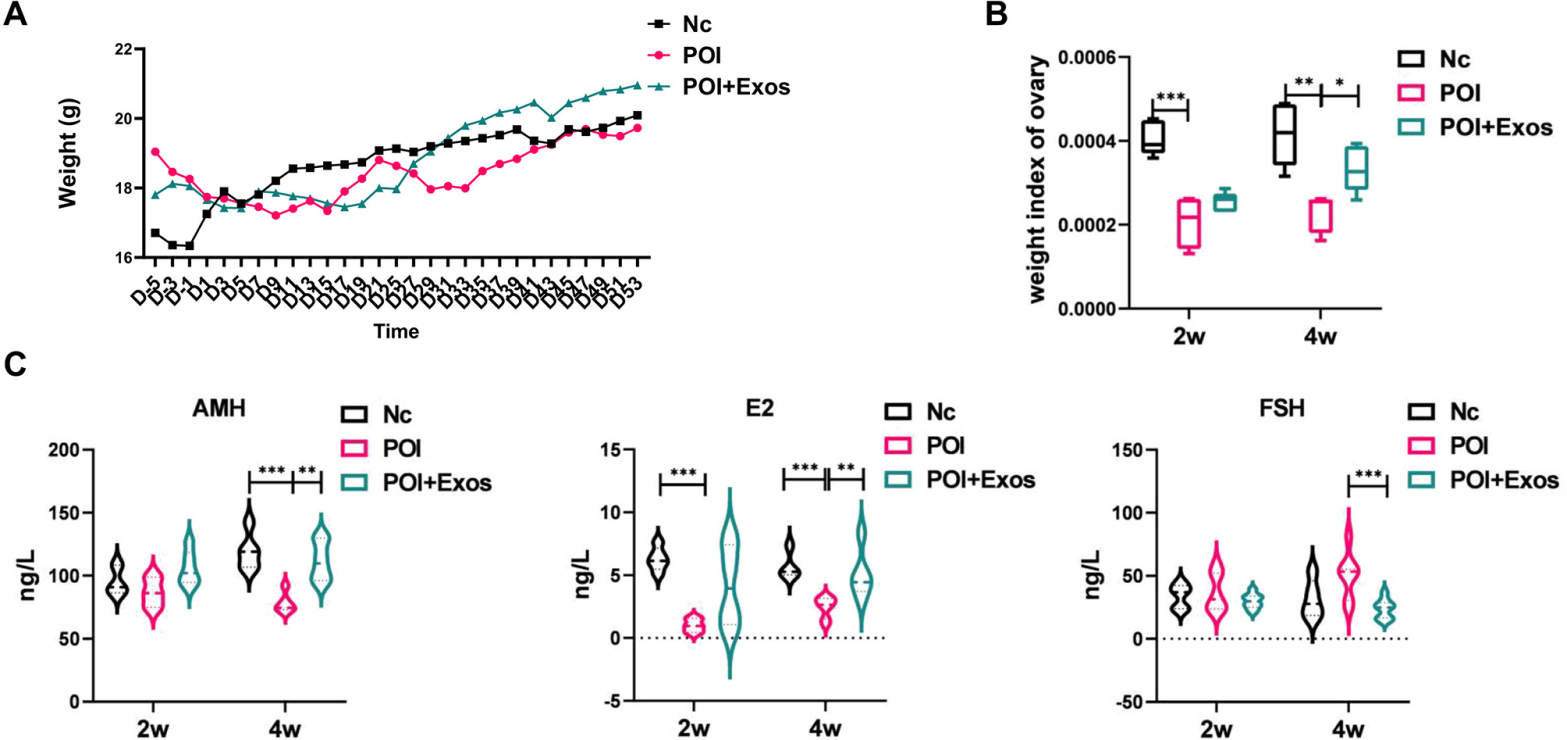
Weight and hormone levels of mice in each group. **A** Changes in body weight among the three groups of mice (Nc group, POI group, and POI + Exos group). **B** Comparison of ovarian weight indices at 2 weeks and 4 weeks post-treatment among the three groups (Nc group, POI group, and POI + Exos group). **C** Comparison of hormone levels among the three groups of mice (Nc group, POI group, and POI + Exos group)

As shown in Fig. 2A, the trend of body weight changes before and after treatment in the three Premature Ovarian Insufficiency mouse groups was essentially the same, with no significant differences between the groups. However, compared to the control group, the ovarian weight index of the POI group significantly decreased at 2 and 4 weeks post-surgery. hUMSC-exos treatment partly rescued the ovaries from the damage caused by chemotherapy drugs. Four weeks after surgery, the ovarian weight index of the POI + Exos group was significantly higher than that of the POI group (Fig. 2B). An ELISA test of ovarian function-related hormones showed that the levels of E2 at 2 and 4 weeks post-modelling and AMH at 4 weeks in the POI group were lower than those in the control group (*P* < 0.05 for all). hUMSC-exos treatment partially restored ovarian function. Four weeks after hUMSC-exos treatment, the levels of AMH and E2 in the POI + Exos group were higher than those in the POI group, and FSH was lower than in the POI group (*P* < 0.05 for all) (Fig. 2C).

### hUMSC-exos reversed CTX-induced reduction in mouse follicle

**Fig. 3:**
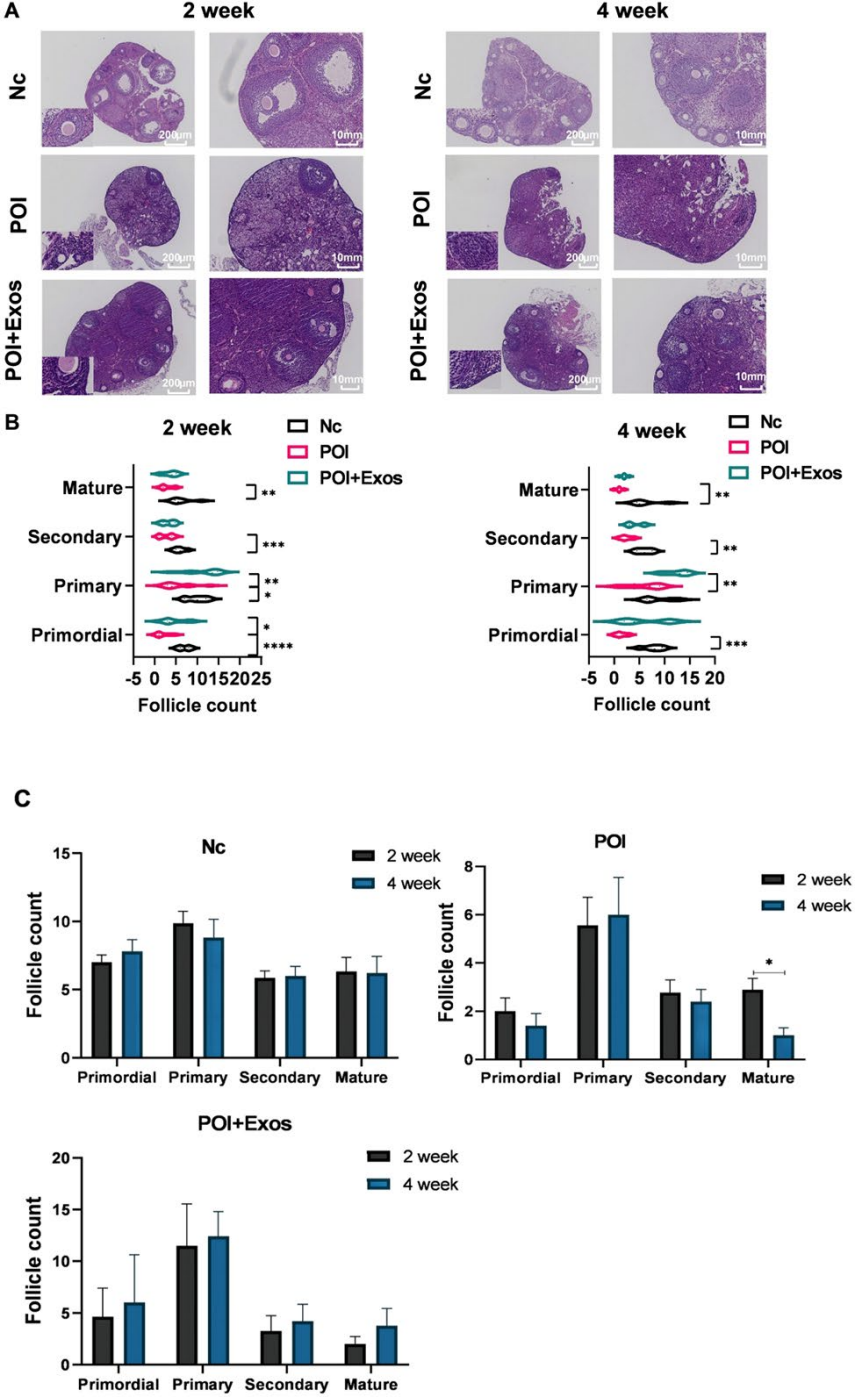
Comparison of follicle numbers among the groups of mice. **A** Hematoxylin and Eosin (HE) staining results of the ovaries in the three groups (Nc group, POI group, and POI + Exos group) at 2 weeks and 4 weeks (magnification 50×, 100X). **B** Statistical chart of the numbers of follicles at each stage in the three groups (Nc group, POI group, and POI + Exos group) at 2 weeks and 4 weeks. **C** Statistical comparison of the number of follicles at different stages between 2 weeks and 4 weeks. (*P < 0.01, **P < 0.01, ***P < 0.001,*n* = 5 of each group)

The results of Hematoxylin and Eosin (HE) staining revealed that under the effects of CTX chemotherapy, mouse ovaries exhibited significant follicular atresia, with a decrease in follicle number that progressed over time (Fig. 3A). Upon comparing the counts of follicles in each group’s sections, the numbers of primordial, primary, secondary and mature follicles in the POI group were significantly reduced at 2 weeks and 4 weeks, compared to the normal group. At 2 weeks, the numbers of primordial and primary follicles in the POI + Exos group were significantly higher than those in the POI group (*p* < 0.05) (Fig. 3B). At 4 weeks, the number of primary follicles in the POI + Exos group significantly increased compared to the POI group. The number of mature follicles was decreased at the fourth week when compared to the second week in the POI group. While, in the POI + Exos group, the number of mature follicles was increased at the fourth week when compared to the second week (Fig. 3C). These results suggest that the in-situ transplantation of exosomes promoted the recovery of ovarian function and physiological function in POI mice, primarily with pre-antral follicles observed in the early stage.

### hUMSC-exos promoted KGN cell proliferation and alleviated CTX-induced KGN cell death

**Fig. 4:**
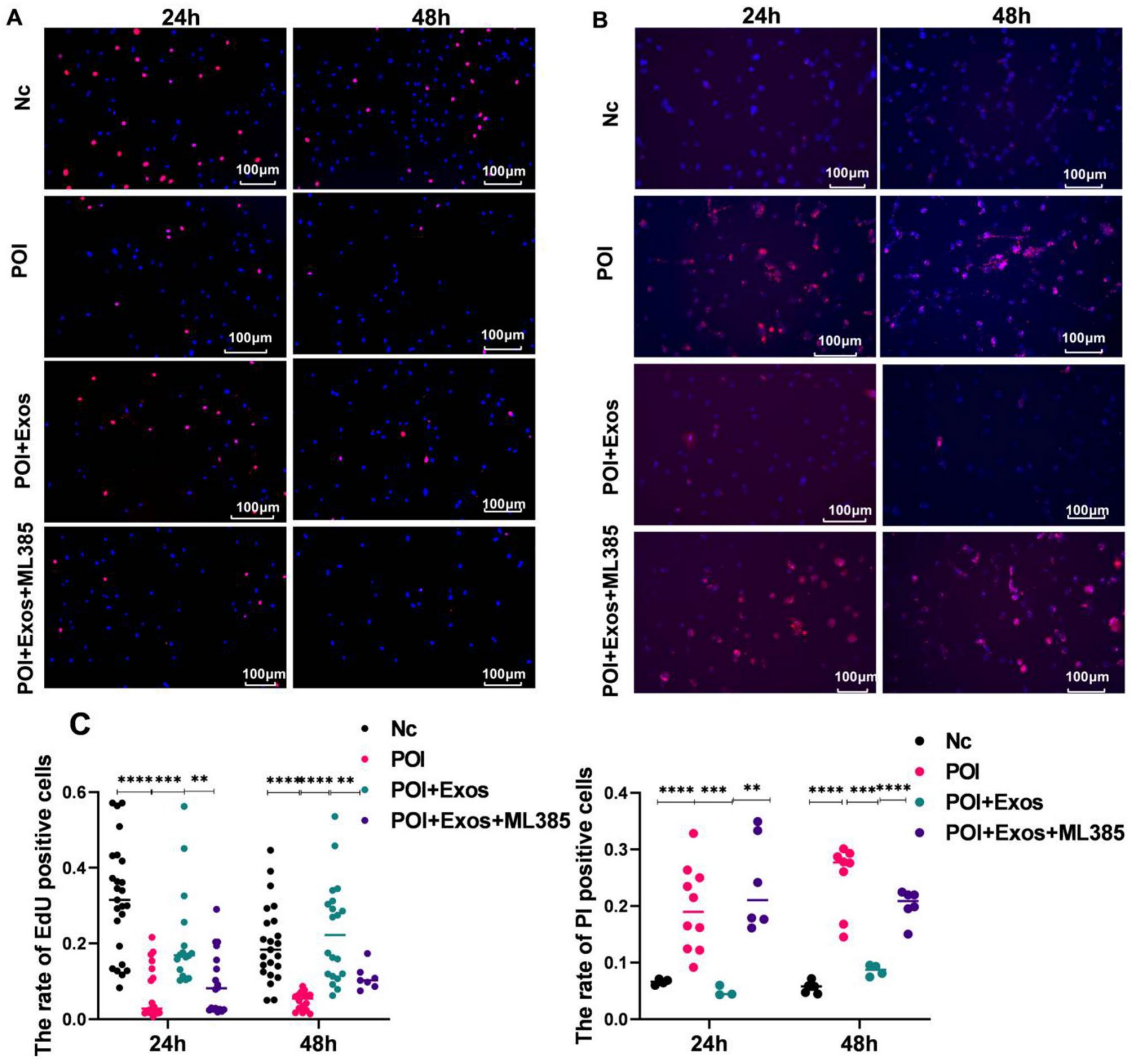
hUMSC-exos alleviated CTX-induced KGN cell apoptosis. **A** Cell proliferation detected by EDU after co-culturing KGN cells treated with hUMSC-exos and CTX for 24 h and 48 h. (Magnification 100×. Red: EDU. Blue: DAPI.) **B** KGN cell death detected by Hoechst/PI after co-culturing KGN cells treated with hUMSC-exos and CTX for 24 h and 48 h. (Magnification 100×. Red: DAPI. Blue: Hoechst.) **C** Statistical results from Fig. 4A and B. **P < 0.01, ***P < 0.001, ****P < 0.001

As depicted in Fig. 4A and C, the proliferation of KGN cells was significantly inhibited after treatment with CTX for 24 and 48 h (*P* < 0.0001). The treatment with hUMSC-exos partly alleviated the inhibition of cell proliferation by CTX. Similarly, the apoptosis of KGN cells significantly increased after treatment with CTX for 24 and 48 h (*P* < 0.0001). The treatment with hUMSC-exos partially reduced CTX-induced cell apoptosis (Fig. 4B and D).

### hUMSC-exos inhibit CTX-induced ferroptosis in KGN cells

**Fig. 5:**
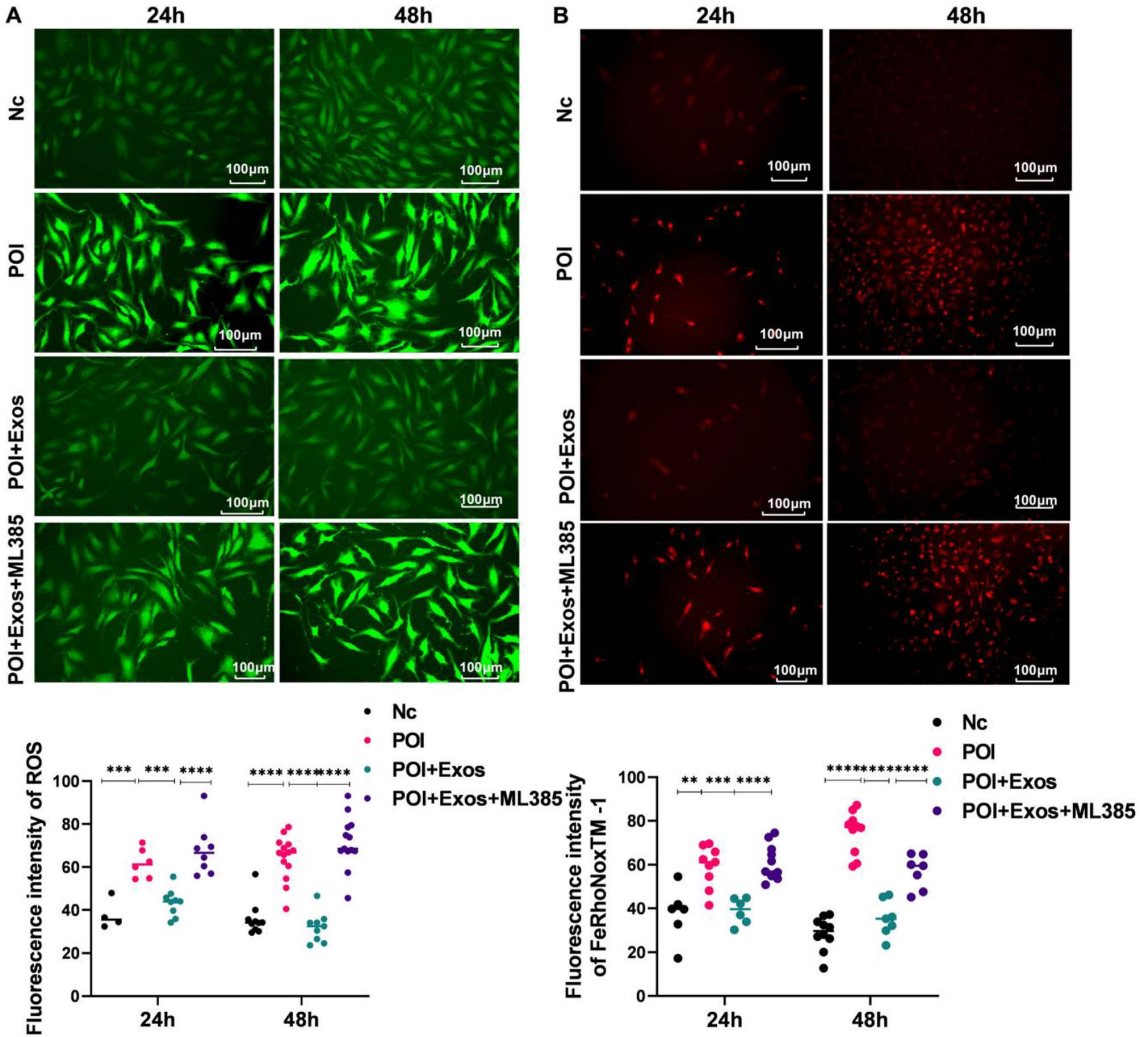
hUMSC-exos alleviated CTX-induced ferroptosis in KGN cells. **A** Comparison of ROS levels in Nc group, POI group, POI + Exos group, and POI + Exos + ML385 group. **B** Detection of Fe2 + in Nc group, POI group, POI + Exos group, and POI + Exos + ML385 group by FeRhoNoxTM − 1 staining. **C** Statistical analysis results of ROS detection and FeRhoNoxTM − 1 staining. (**P < 0.01, ***P < 0.001, ****P < 0.001.)

Compared with the control group, the levels of ROS increased after CTX treatment for 24 h and 48 h (*P* < 0.05). Treatment with hUMSC-exos for 24 h and 48 h reduced the production of ROS in KGN cells induced by CTX (*P* < 0.05). Moreover, treatment with the Nrf2 inhibitor ML385 reduced the inhibitory effect of hUMSC-exos on ROS in KGN cells (*P* < 0.05) (Fig. 5A and C). As detected by FeRhoNoxTM − 1, the trend of Fe^2+^ levels in each group were consistent with that of ROS levels in KGN cells (Fig. 5B and D).

**Fig. 6:**
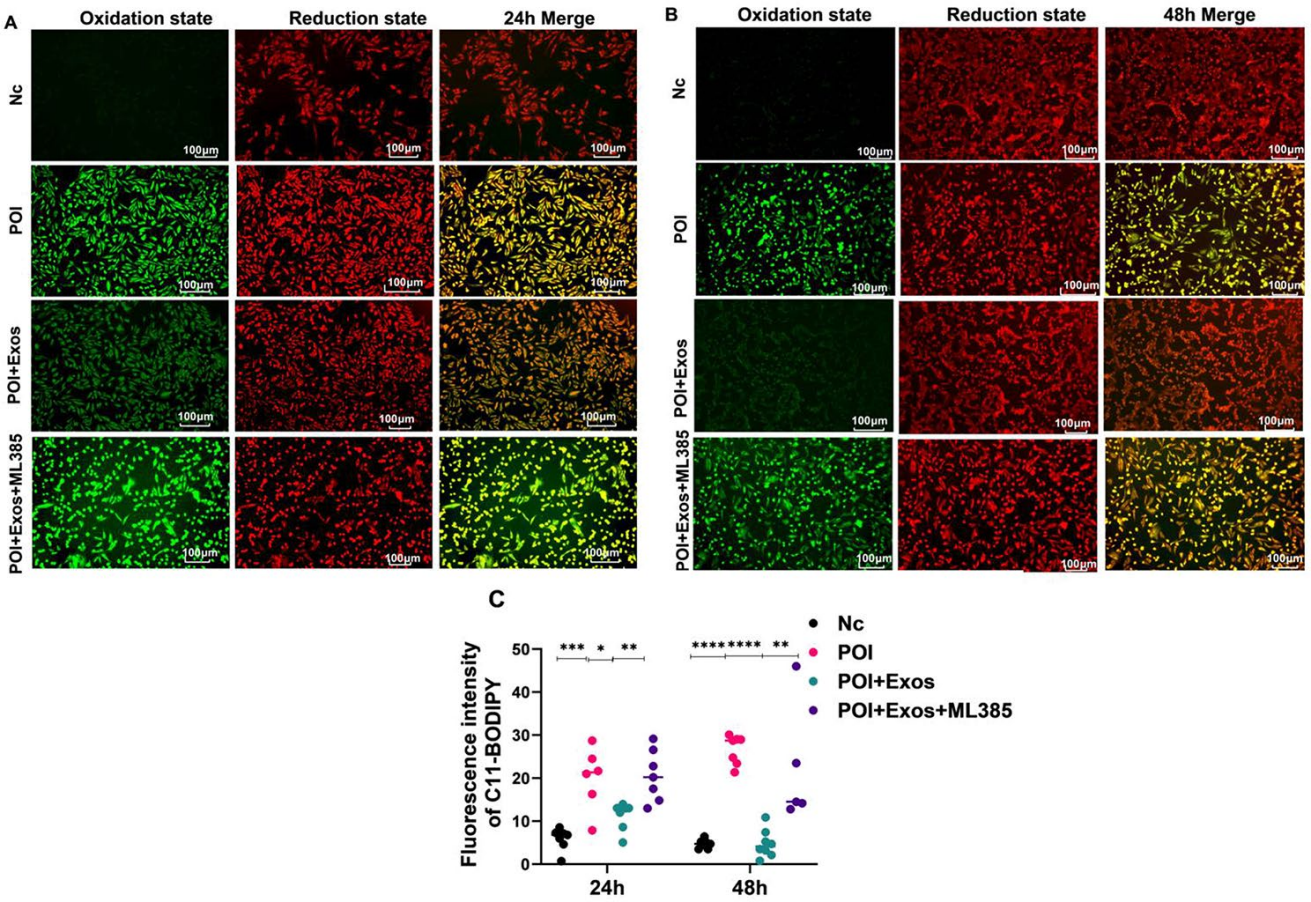
hUMSC-exos alleviated CTX-induced lipid peroxidation in KGN cells. **A** Lipid peroxidation status in Nc group, POI group, POI + Exos group, and POI + Exos + ML385 group after 24 h of treatment. **B** Lipid peroxidation status in Nc group, POI group, POI + Exos group, and POI + Exos + ML385 group after 48 h of treatment. **C** Statistical results of Fig. 6A and B. (*P < 0.05, **P < 0.01, ***P < 0.001, ****P < 0.0001.)

Similarly, CTX treatment for 24 h and 48 h raised lipid peroxidation levels compared to the control group (*P* < 0.05). hUMSC-exos treatment for the same periods reduced these CTX-induced increases (*P* < 0.05). Additionally, the Nrf2 inhibitor ML385 mitigated hUMSC-exos’ suppression of lipid peroxidation in KGN cells (*P* < 0.05) (Fig. 6).

**Fig. 7:**
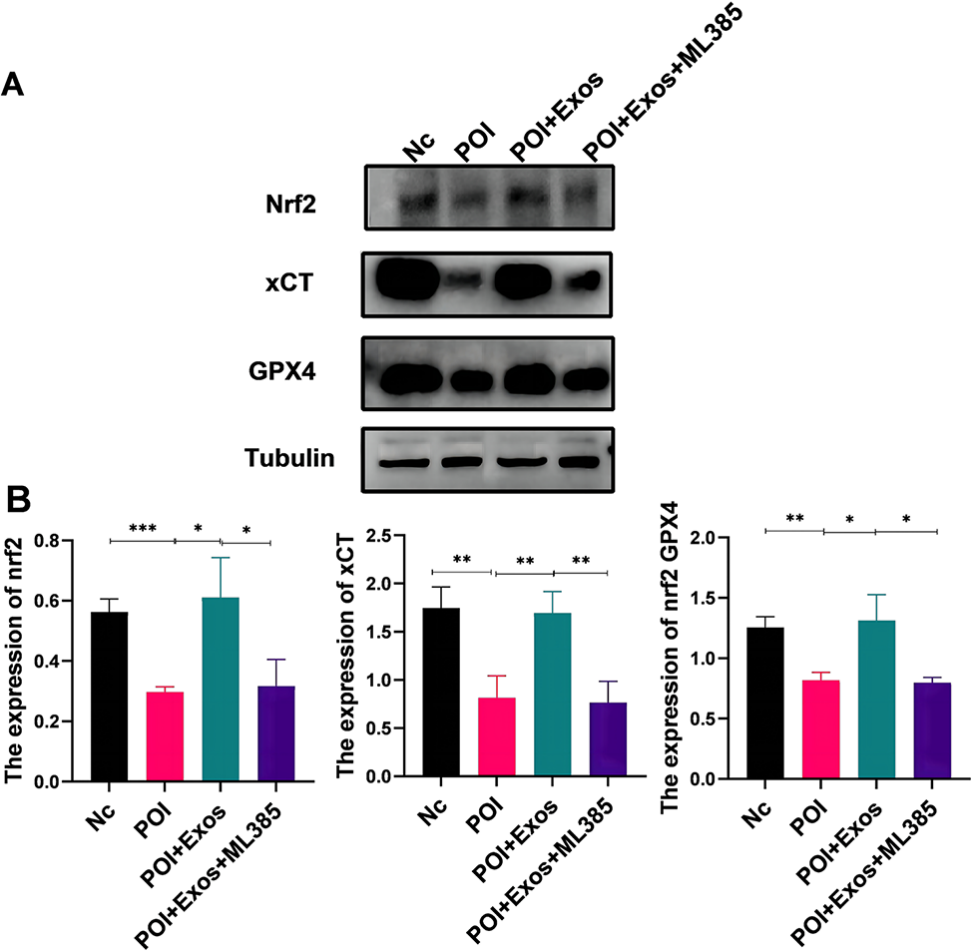
hUMSC-exos Regulate Ferroptosis via the Nrf2/GPX4 Pathway. **A** Western blotting to examine the protein expression levels of Nrf2, GPX4, and xCT in KGN cells 48 h after CTX, CTX + Exos, and CTX + Exos + ML385 treatments. **B** Statistical analysis results of Fig. 7A (*P < 0.05, **P < 0.01, ***P < 0.001)

### hUMSC-exos modulated KGN cell ferroptosis via the Nrf2/GPX4 pathway

Previous studies indicate the Nrf2/GPX4 pathway as a key signal transduction route in ferroptosis. We further examined if hUMSC-exos treatment of POI relates to this pathway. As shown in Fig. 5A, 48 h post-CTX treatment, KGN cells exhibited markedly lowered Nrf2, xCT, and GPX4 protein expressions (*P* < 0.05). Following hUMSC-exos treatment, these protein levels rose (*P* < 0.05). The effect of hUMSC-exos was partially counteracted by the Nrf2 inhibitor ML385 (*P* < 0.05). These findings suggest that hUMSC-exos mitigate CTX-induced ferroptosis via the Nrf2/GPX4 pathway.

## Discussion

Our findings demonstrated that hUMSC-exos alleviate CTX-induced ovarian damage in mice, enhancing ovarian hormone levels and follicles in vivo. In vitro assay showed hUMSC-exos counteracting CTX-induced granulosa cell apoptosis, promoting cellular proliferation and hormone synthesis. Further investigations revealed hUMSC-exos could mitigate CTX-induced reactive oxygen species, iron deposition, and lipid peroxidation. Mechanistically, hUMSC-exos suppressed CTX-induced ferroptosis via the Nrf2/GPX4 pathway, which was reversed by a Nrf2/GPX4 pathway inhibitor. In essence, our study elucidated the role and mechanism of hUMSC-exos in POI through the regulation of ferroptosis, providing critical insights for advancing therapeutic strategies in this field.

Human mesenchymal stem cell therapy is a novel strategy for retain ovarian function and treat female infertility [17]. Available hMSCs include human bone marrow-derived mesenchymal stem cells, human adipose tissue-derived stem cells, and human amniotic membrane mesenchymal stem cells [18, 19]. However, problems such as embolism, immunogenicity, malignant transformation, poor cell engraftment and post-transplantation survival limit the application of stem cell therapy [20, 21]. Notably, exosomes do not express major histocompatibility complex (MHC) I or II [22], which overcomes all the disadvantages of cell therapy [23].

Some studies indicated that certain chemicals have a certain toxic effect on the ovaries of females via increasing oxidative stress in the ovaries to trigger cell apoptosis, thereby affecting the survival of ovarian granulosa cells. The redox balance plays a critical role in regulating follicle growth, angiogenesis, and hormone levels. However, when the ROS-antioxidant balance is disturbed, severe oxidative stress (OS) consequences can occur, reducing oocyte quantity and quality. OS mediates microenvironmental changes in genetic material, signaling pathways, and transcription factors, leading to meiotic abnormalities and mitochondrial loss in apoptotic granulosa cells. Therefore, it is likely to improve POI through controlling level OS [24, 25]. Our study confirmed the critical role of hUMSC-exos in the regulation of intracellular oxidative stress induced by CTX.

Recent research reveals a pivotal role for exosomes in ovarian function protection, offering a novel direction for the prevention and treatment of ovarian diseases [6, 26, 27]. Exosomes function significantly in cellular communication [28, 29], disease progression, and immune regulation, with a particular emphasis on managing oxidative stress. Firstly, exosomes can extracellularly discharge antioxidant proteins, enzymes, and small-molecule antioxidants to eliminate oxidative stress products, thereby safeguarding damaged cells. They are found to encapsulate antioxidant enzymes like Superoxide Dismutase (SOD) and Glutathione Peroxidase (GPX) [30, 31]. Under oxidative stress, these enzymes can be transported to distressed cells via exosomes, curtailing oxidative stress levels and preserving key intracellular biomacromolecules. Secondly, exosomes serve a regulatory role in oxidative stress. Stress conditions elevate the production and release of intracellular exosomes. They can both shield damaged cells by disseminating antioxidant proteins and bolster cellular antioxidant capabilities by stimulating the NF-E2 related factor 2 (Nrf2) signalling pathway, thereby advancing the transcription of antioxidant genes [32, 33]. Additionally, exosomes can modulate oxidative stress responses by activating ROS production pathways in other cells and adjusting intracellular antioxidant levels, influencing cell survival and metabolism [34]. Our study shows that human umbilical cord mesenchymal stem cell-derived exosomes (hUMSC-exos) protected granulosa cells by impeding ferroptosis through the Nrf2/GPX4 pathway.

Additionally, our study highlights the potential of exosomes in impeding chemotherapy-induced ovarian toxicity, laying a robust foundation for devising targeted strategies for ovarian protection. Further research could scrutinize the interplay between exosomes and ovarian cells, elucidating their explicit regulatory functions in preserving ovarian functionality.

We recognized the limitations and prospective trajectories of our study. Despite positive indications from our findings, they require further rigorous assessment and validation prior to clinical application. Future studies could enhance the investigation into the biological attributes and operational mechanisms of exosomes, with the aim to pinpoint more accurate and efficacious treatment strategies, thereby broadening the spectrum of choices for managing chemotherapy-induced premature ovarian failure.

## Data Availability

The authors declare that the data and material of this study are available within the article
